# Optimizing PSMA Radioligand Therapy for Patients with Metastatic Castration-Resistant Prostate Cancer. A Systematic Review and Meta-Analysis

**DOI:** 10.3390/ijms21239054

**Published:** 2020-11-28

**Authors:** Finn Edler von Eyben, Glenn Bauman, Rie von Eyben, Kambiz Rahbar, Cigdem Soydal, Alexander R. Haug, Irene Virgolini, Harshad Kulkarni, Richard Baum, Giovanni Paganelli

**Affiliations:** 1 Center of Tobacco Control Research, DK-5230 Odense M, Denmark; 2 Department of Oncology, Western University, London, ON N6A 5W9, Canada; glenn.bauman@lhsc.on.ca; 3 Department of Radiation Oncology, Stanford University, Stanford, CA 94305, USA; rieve@stanford.edu; 4 Department of Nuclear Medicine, University of Muenster, Albert-Schweitzer-Campus 1, 48149 Muenster, Germany; kamiz.rahbar@unii-muenster.de; 5 Department of Nuclear Medicine, University of Ankara, 06800 Ankara, Turkey; csoydal@yahoo.com; 6 Division of Nuclear Medicine, University Hospital, 1010 Vienna, Austria; alexander.haug@meduniwien.ac.at; 7 Department of Nuclear Medicine, University Hospital Innsbruck, 6020 Innsbruck, Austria; irene.virgolini@tirol-kliniken.at; 8 Department of Molecular Radiotherapy and Molecular Imaging, Zentralklinik, 99438 Bad Berka, Germany; harshad.kulkarni@outloook.de; 9 Theranostics Center, Johan Wolfgang Goethe University Frankfurt/Main, 60437 Frankfurt, Germany; baumr@gmail.com; 10 Istituto Scientifico Romagnolo per lo Studio e la cura dei Tumori (IRST) IRCCS, 7014 Meldola, Italy; giovanni.paganelli@irst.emr.it

**Keywords:** adverse effects, decline of prostate specific antigen, metastases, overall survival, predictive factors, prostate cancer, prostate-specific membrane antigen, theranostics

## Abstract

The aim of the review was to evaluate patient and treatment characteristics for patients with metastatic castration-resistant prostate cancer (mCRPC) treated with PSMA radioligand therapy (PRLT) associated with above-average outcome. The systematic review and meta-analysis followed recommendations by the Preferred Reporting Items for Systematic reviews and Meta-Analysis (PRISMA). We searched for publications in PubMed, Embase, and ClinicalTrials.gov up to 31 September 2020. Thirty-six publications and four duplicates reported 2346 patients. Nearly two-thirds of the patients had bone metastases. Median overall survival (OS) was 16 months. Asymptomatic patients and patients with only lymph node metastases lived longer than symptomatic patients and patients with more extensive metastases. Patients treated with an intensified schedule of ^177^Lu PRLT lived longer than those treated with a conventional schedule. Half of the patients obtained a PSA decline ≥ 50% and these patients lived longer than those with less PSA decline. Approximately 10% of the patients developed hematologic toxicity with anemia grade 3 as the most severe adverse effect. Characteristics for patients, cancer, restaging, and PRLT predict above average overall survival following treatment with PRLT.

## 1. Introduction

Prostate cancer (PC) is the most frequent non-cutaneous cancer among adult men. Whilst most men present with localized cancer, some men who present with or progress to metastatic PC that after initial treatment with androgen deprivation therapy (ADT) eventually progress to a castration-resistant state (mCRPC). Patients with mCRPC are treated with androgen receptor signaling targeted inhibitors, such as abiraterone and enzalutamide, and chemotherapy such as docetaxel and cabazitaxel [1]. More recently, prostate-specific membrane antigen (PSMA) based radioligand therapy (PRLT) has been used.

Patients with endstage mCRPC responded better to treatment with ^177^Lu-PRLT than patients with mCRPC resistant to two lines of established drugs to third-line treatment [2]. A prospective study by Hofman et al showed that PRLT had an impressive response rate and tolerability. A preliminary presentation of a prospective randomized trial, TheraP, NCT03392428, ClinicalTrials.gov, supports that PRLT gives a better outcome than third-line treatment with cabazitaxel [3]. The interest in PRLT is growing. Of 214 publications on PRLT for patients with mCRPC listed in PubMed September 2020, 66 (31%) are published in 2020.

Many publications reported factors associated with the outcome after PRLT [4]. So the present systematic review and meta-analysis aimed to analyze patient and treatment characteristics associated with an above-average overall survival (OS). The systematic review also aimed to determine the proportion of patients with severe adverse effects (SAE).

## 2. Results

### 2.1. Overall Findings

**Figure 1:**
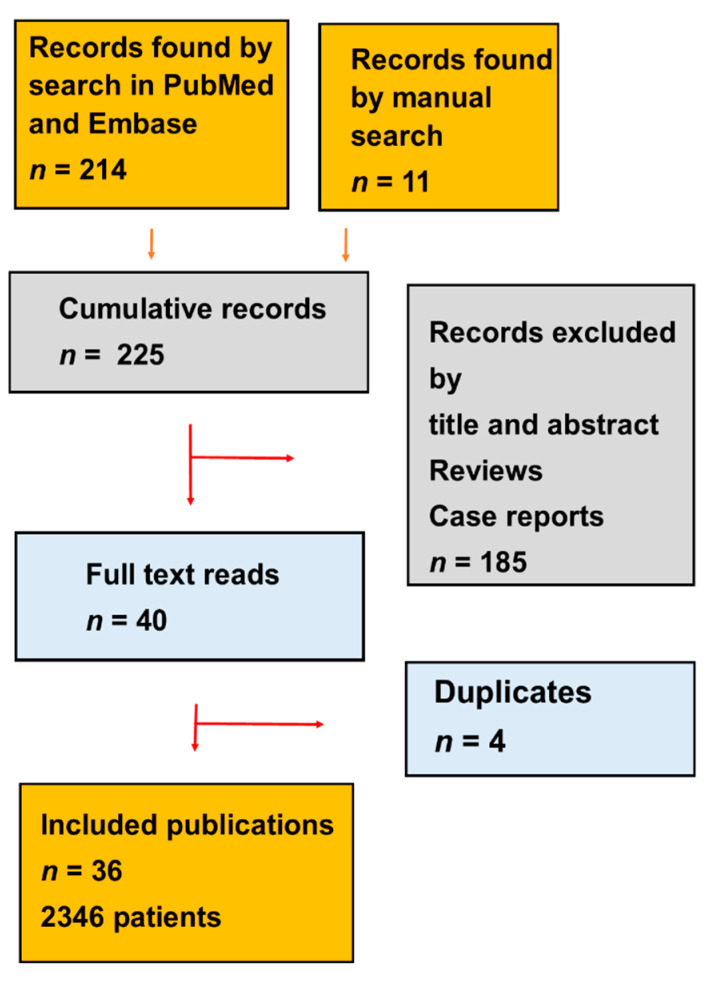
The PRISMA (The Preferred Reported Items in Systematic review and Meta-Analysis) flow diagram shows the selection process in the systematic review.

The search for literature gave 225 records. 36 original research publications with 2346 patients and four duplicates met our eligibility criteria, as shown in Figure 1 and Table 1A,B [4,5,6,7,8,9,10,11,12,13,14,15,16,17,18,19,20,21,22,23,24,25,26,27,28,29,30,31,32,33,34,35,36,37,38,39,40,41,42,43]. Most publications reported retrospective studies and four publications reported prospective studies [21,22,23,26]. Restaging in most publications was carried out with PSMA PET/CT and one publication [21] used both 2-deoxy-2-[^18^F]-D-glucose (FDG) and PSMA PET/CT. Most publications reported ^177^Lu PRLT and five publications reported ^255^Act PRLT [10,16,23,25,33].

Most ^177^Lu based publications used ^177^Lu PSMA-617 and one publication used ^177^Lu PSMA I&T [26]. Most publications reported a conventional schedule for ^177^Lu PRLT using 6 GBq ^177^Lu for each cycle of PRLT and ≥8 weeks between cycles, and three publications reported an intensified schedule [14,20,28]. Most publications reported the outcome after the first series of PRLT whereas five publications reported outcome after a second series of PRLT after relapse after the first series [10,16,21,24]. Four duplicates added relevant data to the findings reported in the original research reports [4,5,18,19].

### 2.2. Bias

**Figure 2:**
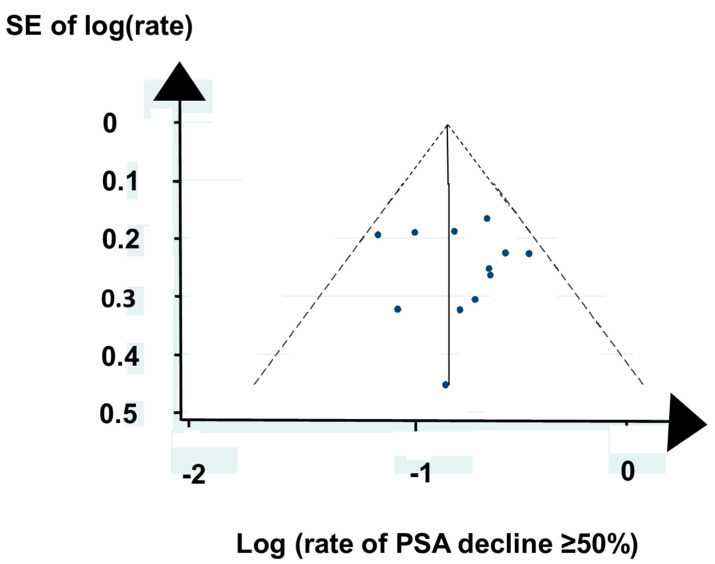
A Funnel plot of publications regarding PSA decline ≥ 50% with first series of ^177^Lu PRLT indicates no evidence of bias.

The selected publications had aspects that could have caused bias. Some publications did not state whether they reported consecutive patients, other publications reported preliminary results, and a third group of publications did not report on all outcomes our systematic review aimed to address. Nevertheless, a Funnel plot of the rate of PSA decline ≥ 50% after PRLT in publications of the first series of ^177^Lu PRLT did not reveal significant asymmetry or outliers, as shown in Figure 2.

Publications of retrospective and prospective studies did not differ significantly regarding rate of PSA decline ≥ 50%. Therefore, our systematic review based the summaries and analyses on all selected publications.

### 2.3. Patients

Nearly two-thirds of the patients had bone metastases. Two patient characteristics were significantly associated with the OS after PRLT, as shown in Table 2. Chemotherapy-naïve patients lived longer than chemotherapy-resistant patients [6,27,31,37]. Asymptomatic patients (performance status (PS = 0)) [4,6,22] lived longer than symptomatic patients (PS = 1–2). Patients with mCRPC resistant to androgen receptor pathway inhibitors and cabazitaxel in the TheraP trial [44] who were randomized to PRLT had a higher PSA decline ≥ 50% than the patients with end-stage mCRPC given PRLT in the previous systematic review (66% vs. 44%) [2]. Patients with only one kidney [39] tolerated treatment with PRLT.

### 2.4. Cancer

Four characteristics of mCRPC had an impact on OS after PRLT. Nearly two-thirds of the patients in the selected publications had bone metastases Patients with extensive bone marrow metastases [9] tolerated treatment with PRLT. More patients with lymph node metastases (LNM) had PSA decline ≥ 50% after PRLT than patients with bone metastases (36 of 45 versus 38 of 100, *p* < 0.0005, χ^2^ test) [30,37]. Patients with bone metastases and lung metastases lived longer than patients with liver metastases [5,11,31]. Patients with a small total tumor volume lived longer than patients with a large total tumor volume [19]. Patients who had cancer lesions with a high uptake of ^177^Lu lived longer than patients with a low uptake [23,45].

Patients with normal serum tumor markers lived longer than patients with raised serum tumor markers [24]. Patients with a normal serum alkaline phosphatase lived longer than those with a raised serum alkaline phosphatase, as shown in Table 2.

### 2.5. Restaging

**Figure 3:**
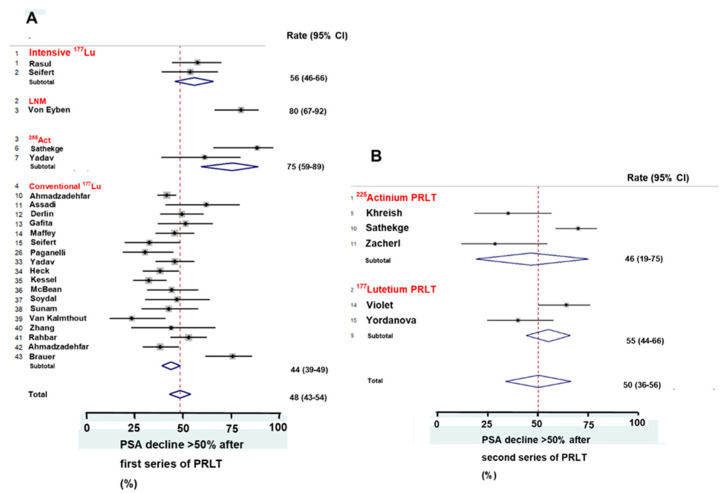
Forest plots show the rate of PSA decline ≥ 50% following two series of PRLT (PSMA based radioligand therapy) using either ^225^Act or ^177^Lu as radionuclide. The rate of PSA decline ≥ 50%was grossly similar after the first series of PRLT (**A**) and after the second series of PRLT (**B**).

The characteristic of the restaging PET/CT had an impact on the OS after PRLT. Most publications carried out restaging before PRLT with the use of only PSMA PET/CT. One publication carried out restaging with both FDG and PSMA PET/CT [21]. More patients in this publication had a PSA decline ≥50% than the patients in publications restaged with only PSMA PET/CT, as shown in Figure 3B. Patients with a high average Standard Uptake Value (SUV_average_) and a high minimal SUV (SUV_min_) in PSMA-avid tumor lesions lived longer than patients with lower SUV_average_ and lower SUV _min_ [28].

### 2.6. Radioligand

Three characteristics of the PRLT had an impact on the OS. Patients treated with ^177^Lu PSMA-617 and ^177^Lu PSMA I&T [30] had similar rates of PSA decline ≥ 50%. More patients treated with an intensive schedule for ^177^Lu PRLT in the first series had a PSA decline ≥ 50% than those treated with a conventional schedule, as shown in Figure 3A. Surprisingly, in the second series of PRLT, ^177^Lu PRLT gave a higher rate of PSA decline ≥ 50% than ^225^Act PRLT, as shown in Figure 3B. More patients treated with a full dose of ^225^Act PRLT in the second series had PSA decline ≥ 50% than those treated with a tandem of both ^255^Act and ^177^Lu PRLT.

Treatment of the relapse differed for patients who had responded to the first series of PRLT and later relapsed [21,37]. Relapsing patients treated with a second series of PRLT lived longer than patients treated with established drugs [37,38]. Violet et al. [21] showed that patients treated with a second series of PRLT lived longer than all patients in their study (26 months vs. 13 months). Of patients with LNM, patients given a cumulative ^177^Lu activity ≥ 18 GBq lived longer than patients given a lower cumulative ^177^Lu activity [37].

Treated with ^255^Act PRLT, more patients had PSA decline ≥ 50% after the first series of PRLT than patients treated with ^177^Lu PSMA-617 and ^177^Lu PSMA I&T. But the ^225^Act findings were heterogeneous, as shown in Figure 3A,B. Sathekge et al. [16] used a higher administered ^255^Act activity per cycle (initially 8 MBq per cycle) than Khreish et al. [10] (initially median 5.3 MBq per cycle).

### 2.7. Response

Both for the first and the second series of PRLT, half of the treated patients with mCRPC had a PSA decline ≥ 50%, as shown in Figure 3A,B. Fourteen publications with 1266 patients reported the rate of PSA decline ≥50% [6,12,14,20,21,22,30,31,34,37,39,41,42,43]. Soydal et al [33] showed that patients with a PSA decline < 50% and patients with PSA progression after PRLT had similar OS. Overall, patients with PSA decline ≥ 50% lived longer than those with less PSA decline (median 20 months vs. 12 months, *p* = 1.6 × 10^−6^, Fisher’s test) [21,30,31], as shown in Table 2.

### 2.8. Survival

**Figure 4:**
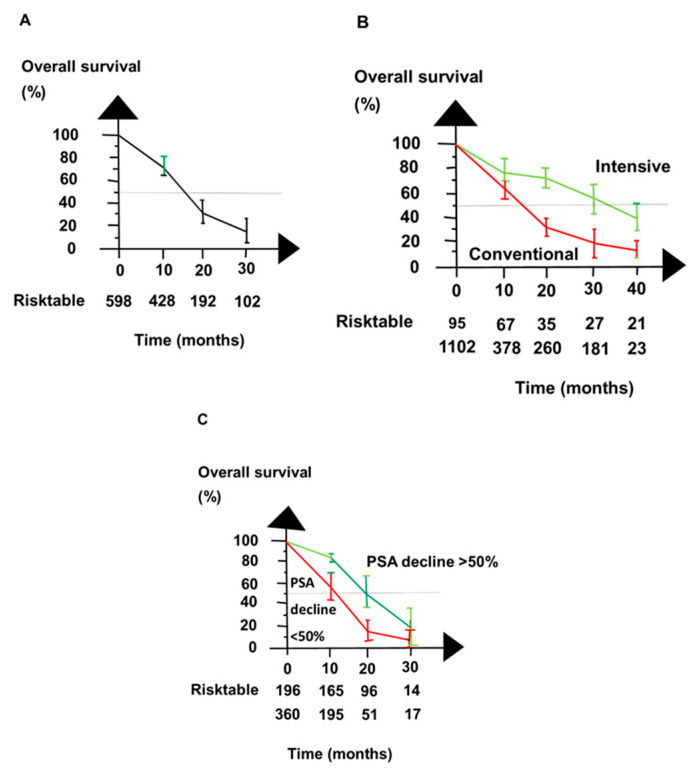
Overall survival for patients with mCRPC reported in publications of the first series of ^177^Lu PRTL was encouraging. (**A**) shows the publications had consistent overall survival. (**B**) shows that patients given ^177^Lu PRLT with an intensive schedule (green line) lived longer after PRLT than patients given ^177^Lu PRLT with the conventional schedule (red line). (**C**) shows that patients with PSA decline ≥ 50% after PRLT (green line) lived longer than patients with PSA decline < 50% (red line).

Median OS was 16 months regarding patients in publications treated with the first series of ^177^Lu PRLT apart from the publications of patients with only LNM [36] and only liver metastases [17], as shown in Figure 4A. Patients treated with an intensified schedule of ^177^Lu PRLT lived longer than patients treated with a conventional schedule, as shown in Figure 4B. Patients who had a PSA decline ≥ 50% lived longer than those with less PSA decline, as shown in Figure 4C. Intensified PRLT had a more long-lasting impact on OS than PSA decline ≥ 50% had.

LNM patients in two publications had a 2-years OS of 100% [4,37], and LNM patients in a third publication lived longer than patients with more extensive metastases [6]. Patients with bone and lung metastases lived longer than patients with liver metastases [5,30,31]. So, for patients with visceral metastases, the determining site of the metastases was important for OS.

Regarding ^255^Act PRLT, the patients reported by Sathekge et al. [16] lived longer than the patients reported by Khreish et al. [10] (18 months vs. 12 months).

### 2.9. Adverse Effects

**Figure 5:**
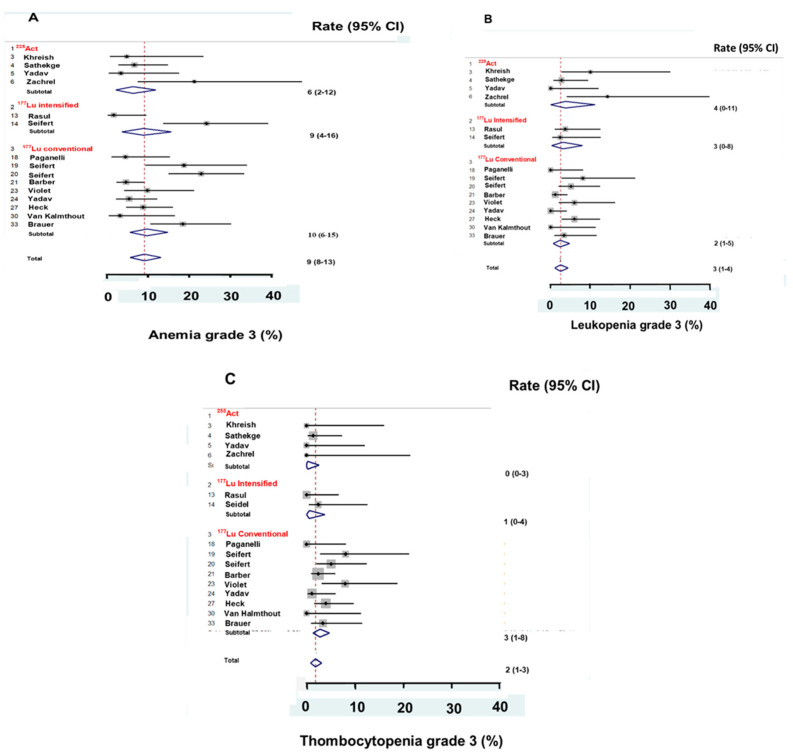
^225^Actinium PRLT and intensified and conventional schedule of ^177^Lu PRLT gave similar hematologic adverse effects grade 3. More patients had anemia grade 3 (**A**) than leucopenia grade 3 (**B**) and thrombocytopenia grade 3 (**C**).

Treatment with ^177^Lu PRLT was safe. None of the patients died of severe adverse effects (SAE) and none of the patients developed leukemia. Some patients discontinued planned treatment with PRLT mainly due to the PC had progressed. Fourteen publications with 844 patients reported SAE [10,13,14,16,17,20,21,22,25,27,36,43], Severe adverse effects after PRLT were rare and mainly hematologic adverse effects grade 3. Of the treated patients, a median of 10% had anemia grade 3, median 3% had leucopenia grade 3, and median 2% had thrombocytopenia grade 3, as shown in Figure 5A–C. The patients had similar rates of grade 3 hematologic adverse effects whether PRLT was administered as ^225^Act PRLT or as ^177^Lu PRLT with an intensified or a conventional schedule. Less than 1% of the patients had hematologic adverse effects grade 4 and severe non-hematologic adverse effects.

Renal toxicity grade 3 was reported for 1 of 43 patients in the publication by Yordanova et al. [24] and in 0 of 43 patients in the publication by Paganelli et al. [13] and for 0 of 167 patients in the publication by Barber et al. [27]. Fatigue grade 3 was reported for 1 of 50 patients in the publication by Violet et al [21] and for 0 of 14 patients in the publication of Zacherl et al. [25] and for 0 of 100 patients in the publication by Heck et al. [30]. Xerostomia was reported for 0 of 14 patients in the publication by Zacherl et al. [25] and for 0 of 17 patients in the publication by Sathekge et al. [33].

## 3. Discussion

**Figure 6:**
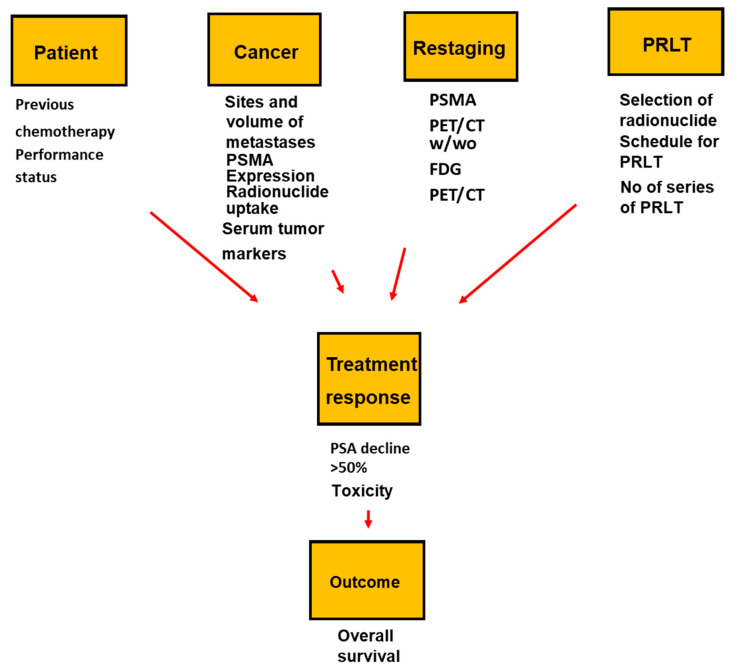
Conceptual model for factors impacting response and overall survival as patients with mCRPC are treated with PSMA based radioligand therapy (PRLT).

^177^Lu PRLT is effective and safe. Our systematic review showed that characteristics regarding patients, cancer, restaging, and PRLT contribute to an above-average OS after PRLT of patients with mCRPC, as summarized in Figure 6. The findings were reproducible, marked, and highly significant. ^177^Lu PRLT gave a low rate of severe adverse effects irrespective of the studies of ^177^Lu PRLT used a conventional or intensified schedule. A Funnel plot did not indicate the publications had a significant bias.

Like our systematic review, another recent systematic review showed that patients with visceral metastases treated with PRLT lived shorter than patients with bone metastases [45]. Additionally, the Prostate Cancer Working Group for reporting studies 3 (PCWG3) [46] considers visceral metastases to be a separate late phase in the progression of mCRPC. Our systematic review adds that only hepatic metastases caused the negative impact visceral metastases to have an outcome after PTRLT relative to that of bone metastases.

Our findings add information regarding the use of ^177^Lu PRLT to that of guidelines by the European Association of Nuclear Medicine (EANM) [47]. Furthermore, our systematic review evaluated the effects and SAE with ^225^Act PRLT.

Regarding patient characteristics, chemotherapy-naïve patients were treated at an earlier phase in the sequence of treatments of mCRPC than patients resistant to chemotherapy. Cancer lesions in chemotherapy-naive patients might be more homogeneous than cancer lesions in chemotherapy-resistant patients. For many cancers in addition to PC, asymptomatic patients with good performance status live longer than patients with symptoms and poor performance status.

The site and extent of mCRPC were important for the outcome after PRLT. Patients with only lymph node metastases may represent a more homogeneous cancer population compared with patients with bone and visceral metastases. That in part explains why patients with LNM have an especially good OS after PRLT [5,37]. Our systematic review evaluated restaging before treatment with PRLT. Previous systematic reviews summarized restaging with PSMA PET/CT in general [48,49]. PSMA PET/CT resulted in a better staging of patients with PC than conventional imaging such as bone and CT scans [50,51].

On restaging PSMA PET/CT, a high SUV_average_ and a high SUV_min_ of ^68^Ga in PSMA-avid tumor lesions were associated with a long OS after PRLT [18]. A high ^68^Ga uptake in PSMA PET/CT scans may be associated with a high ^177^Lu uptake in patients given PRLT and the high ^177^Lu uptake will expose cancer lesions for a high radiation dose [50].

Adding FDG PET/CT to the restaging PSMA PET/CT before PRLT helps oncologists to select patients with a high PSMA homogeneity. Patients with discordant FDG and PSMA PET/CT findings had an extremely poor OS of 2.5 months [52]. Further patients with cancer lesions without FDG uptake had the best prognosis [53]. However, in our systematic review, many patients who underwent restaging with only PSMA PET/CT responded objectively to PRLT and had a longer OS.

^225^Actinium, an alpha emitter, may be more effective in PRLT than ^177^Lutetium, a beta emitter. More patients in the publication by Sathekge et al. [16] had PSA decline ≥ 50% than the patients in the publication by Kratochwil et al [54] (60/73 (80%) vs. 23/38 (63%)). Surprisingly, publications on ^255^Act PRLT in our systematic review did not show a clear trend in favor of ^225^Act PRLT compared with ^177^Lu PRLT as second-line treatment after failure to the first series of PRLT.

As expected, patients treated with ^177^Lu PSMA I&T and ^177^Lu PSMA-617 had a similar outcome. The similarity reiterates that the beta particles of ^177^Lu PRLT are effective to cause the death of cancer cells irrespective of the ligand in the radioligand.

Our systematic review adds important information regarding PRLT. Interestingly, increased ^177^Lu activity in a cycle of ^177^Lu PRLT and a shortened interval between the cycles improved the efficacy of PRLT without increased SAE. A publication reported a study [55] that increased ^177^Lu activity in PRLT up to 9 GBq per cycle without increased severe adverse hematologic effects.

A PSA decline of ≥50% after PRLT was associated with an above-average OS. The association is consistent with PCWG3 recommendations [46]. It is also consistent with a general trend in oncology. Patients who obtain a partial response from chemotherapy live longer than patients who obtain only no change or progressive disease. But the intensified schedule for PRLT had a more long-lasting impact on OS than the level of PSA decline, as shown in Figure 4.

Both serum PSA and repeat PSMA PET/CT may be used in monitoring response to PRLT [15]. Most often the two variables show concordant findings. Furthermore, for patients with rising serum PSA without progression on PSMA PET/CT during follow-up, the discordance may be due to the progression of cancer elements not expressing PSMA. For patients with progression on PSMA PET/CT without a rise of serum PSA, the discordance might be due to the progression of cancer elements not producing PSA.

The World Association of Radiopharmaceuticals and Molecular Therapy (WARMTH) study [6], the survival advantage for patients with only LNM remained during four years of follow-up whereas the previous history regarding chemotherapy had limited impact on OS in the fourth year of follow-up. Complementarily, our systematic review showed a positive impact on OS from intensified ^177^Lu PRLT remained during four years of follow-up.

Regarding toxicity, nearly all patients tolerated ^177^Lu PRLT. Surprisingly, the selected publication on ^225^Act PRLT did not report more SAE than the publication on ^177^Lu PRLT. For comparison established drugs for mCRPC relatively commonly gave rise to grade 3 cardiovascular events that caused discontinuation of the treatment. [56] Furthermore, in the PREVAIL trial [57], 0.6% (5/800) of the patients treated with enzalutamide had drug-induced epileptic seizures so the treatment was discontinued for this subgroup of patients.

Ongoing trials may validate the findings of our systematic review as many of the ongoing trials investigate PRLT as monotherapy. Other reviews summarized ongoing trials of PRLT for PC registered at ClinicalTrials.org [58,59]. A recent review reported more details regarding the ongoing trials [60]. Three publications described the design of three trials in detail [44,61,62]. Some trials examine whether patient characteristics may have an impact on outcome with PRLT (NCT 03454750, NCT03828838, and NCT03511664, ClinicalTrials.org).

In the treatment of mCRPC, PRLT is an optimal candidate for being combined with established drugs. Trials examine whether adding PRLT to the established drugs enzalutamide and docetaxel increases response and outcome: ENZA-p, ANZUP 1901, and NCT04343885, ClinicalTrials.org. Two trials examine a combination of the monoclonal antibody against the programmed death receptor 1 (PD1), pembrolizumab, and PRLT: NCT03658447, and NCT03805594. ClinicalTrials.org. One trial, LuPARP, NCT 03874884, examines a poly(ADP-ribose) polymerase (PARP) inhibitor Olaparib combined with PRLT.

Our systematic review has limitations. It reports only a few patients with favorable patient characteristics, only a few patients treated with intensified ^177^Lu PRLT, and only two radioligands used as monotherapy for patients with mCRPC. Our systematic review did not report the combined effect of all characteristics that determine the response after PRLT and OS.

In conclusion, characteristics of patients, cancer, restaging, and PRLT were associated with an above-average OS after treatment with PRLT. Approximately 10 percent of the patients had severe hematologic adverse effects irrespective of whether the patients had been treated with a conventional and intensified dosage of ^177^Lu PRLT. Oncologists can use the findings to optimize patient selection, predict treatment outcomes, and improve the effect of PRLT.

## 4. Material and Methods

### 4.1. Hypothesis

The null hypothesis regarding PRLT for patients with mCRPC was that neither characteristics of patients nor characteristics of PRLT predict OS and SAE.

### 4.2. Search Strategy

The systematic review followed recommendations by the Preferred Reporting Items for Systematic reviews and Meta-Analysis (PRISMA) [63]. A Pubmed search used MESH terms and free text words ((prostate neoplasm * OR prostate cancer) AND (* lutetium radioligand therapy OR * Lu radioligand therapy OR * Lu PSMA I&T OR * Lu-PSMA-617 OR *Actinium RLT OR RLT) AND (overall survival OR OS)). Two reviewers, GB and FEvE, carried out a similar search in the Embase database and searched for ongoing studies in ClinicalTrials.gov.

The two reviewers searched for publications up to 31 September 2020, as shown in Figure 1. We examined whether the titles and abstracts of the records fulfilled the inclusion criteria. All publications had to report PSA decline or OS. The systematic review included all types of study design as well as both printed publications and publications published ahead of print.

Our systematic review included only original research publications that used small molecule inhibitors of PSMA linked with ^177^Lu or ^225^Act. Furthermore, we included duplicates that added important information to that of the original research publications [4,5,18,19]. We included only publications reporting > 10 patients and restricted language in the publications to English, French, and German. We excluded publications of animal studies, abstracts, case reports, reviews, publications not reporting outcome after PRLT, and most duplicates.

As we read the full text of the selected publications, we applied specified criteria for patients, interventions, comparisons, outcomes, and studies (PICOS). Patients (P) should be more than 18 years, should have multi-resistant mCRPC, and should fulfill guidelines for treatment with PRLT [47]. Site of metastases was classified according to the organ with the most advanced dissemination and worst prognosis. Interventions (I) should be either ^177^Lu PRLT or ^225^Act PRLT. Comparative analyses (C) evaluated whether characteristics differed in impact on the outcome.

The schedule for PRLT was such a characteristic. ^177^Lu PRLT was most often administered in a schedule of 6 GBq per cycle repeated at ≥ 8 weeks intervals. We denoted this schedule as “conventional”. ^177^Lu PRLT was administered in cycles with 7.5 GBq per cycle at 6 weeks intervals and in cycles with 7.4 GBq at 4 weeks intervals in two publications [14,21]. We these schedules as “intensified”.

The principal outcome (O) was OS after PRLT. PCWG3 [46] recommends that reports on outcomes of trials include PSA decline ≥ 50% so we evaluated PSA decline ≥ 50% as a secondary outcome. Adverse effects were reported graded according to the Common Terminology of Clinical Adverse Effects (CTCAE) version 4. Our systematic review defined grade 3 and 4 adverse effects as SAE. The systematic review included publications of retrospective and prospective single-arm cohort studies (S).

The two reviewers independently searched for publications and extracted clinical data from the publications. A third reviewer (CS) could solve discrepancies between the two reviewers. For each publication, we registered the number of patients, median/mean age at the start of PRLT, initial treatment, systemic treatments before PRLT, and median/mean PSA levels at the start of PRLT, as shown in Table 1. We also registered the radionuclide, median activity per cycle of PRLT in the first series of PRLT, the median interval between cycles, rate of PSA decline ≥ 50%, and treatment after failure to the first series of PRLT.

For outcomes, we gave priority to characteristics which two or more publications reported as being significant for OS and to characteristics that publications pointed out as significant in multifactorial analyses. We registered OS specifically at 10, 20, and 30 months post-PRLT from Kaplan–Meier plots in the publications. Further, we registered a PSA decline ≥ 50% and hematologic and non-hematologic SAE.

### 4.3. Statistical Analysis

The systematic review assessed heterogeneity between publications using χ^2^ tests of OS. We evaluated the risk of bias in a Funnel plot. The systematic review summarized proportions in the publications in Forest plots with the use of the Metaprop command for STATA. Calculations were based on a random effect model, a Freeman–Turkey double inverse transformation, and the Score method. We also used χ^2^ tests as we compared proportions in the publications.

Meta-analyses of OS were carried out manually on Kaplan–Meier plots specifically at 10, 20, 30, and 40 months in Kaplan-Meier plots in the publications according to the method of Parmar et al. [64]. Our systematic review carried out meta-analyses of *p* values according to the Fisher combined probability test [65] and considered *p* values < 0.05 as significant. We carried out the statistical analyses with the Stata 14.2 software (Stata Corp, College Station, TX, USA).

## 4.4. Ethical Approval

All patients in the publications had given informed consent to restaging imaging with PSMA PET/CT, to therapy with PRLT, and evaluation and publications of the findings.

## Figures and Tables

**Table 1:** (**A**) Characteristics in the publications. (**B**). Characteristics in the publications.

**(A)**							
**Author**	**No of Patients**	**Patient Characteristics**					
		Median age (years)	Sites of metastases				
			LN	Bones	Lungs	Liver	Other
Ahmadzadehfar [6]	416	72	30	221	69	87	10
Assadi [7]	25	70	NR	NR	NR	NR	NR
Derlin [8]	71	72	24	39	3	9	0
Gafita [9]	43	72	0	33	5	5	0
Khreish [10]	20	72	0	14	2	2	0
Khreish [11]	28	NR	0	0	0	28	0
Maffey [12]	32	NR	5	24	1	2	0
Paganelli [13]	43	73	1	28	7	7	0
Rasul [14]	54	72	8	37	4	5	0
Rathke [15]	100	70	0	65	12	11	0
Sathekge [16]	73	69	7	60	2	4	0
Seifert [17]	31	73	0	0	0	31	0
Seifert [20]	78	71	0	46	14	18	0
Violet [21]	50	71	2	38	5	5	0
Yadav [22]	90	70	1	78	3	3	5
Yadav [23]	28		1	21	3	3	0
Yordanova [24]	137	71	4	81	28	24	0
Zacherl [25]	14	75	0	10	3	1	1
Aghdam [26]	14	70	0	11	0	1	2
Barber [27]	167	70	19	102	18	18	10
Grubmuller [28]	38	72	8	24	3	3	0
Gupta [29]	22		NR	NR	NR	NR	NR
Heck [30]	100	72	3	62	17	18	0
Kessel [31]	54	72	0	51	24	24	0
McBean [32]	50		0	41	4	5	0
Sathekge [33]	17	65	3	12	1	0	1
Soydal [34]	30	68	NR	NR	NR	NR	NR
Suman [35]	40	63	8	23	0	9	0
Van Kalmthout [36]	30	70	NR	NR	NR	NR	NR
Von Eyben [37]	45	61	45	0	0	0	0
Yordanova [38]	30	72	0	23	4	3	0
Zhang [39]	16	65	2	11	1	1	0
Kesavan [40]	22		2	12	1	2	0
Rahbar [41]	104	70	0	70	18	16	0
Ahmadzadehfar [42]	100	NR	0	66	18	16	0
Brauer [43]	59	72	0	30	9	20	0
Tot no patients	2346		176	1342	286	399	40
Percentage			8	60	12	18	2
**(B)**							
**Author**	**Characteristics of Patients**	**Radioligand Therapy**		**Outcomes**			
	Median PSA (ng/mL)	Dose per cycle (GBq)	Interval between cycles (weeks)	PSA decline > 50% (%)		Median OS (months)	
Ahmadzadehfar [6]	215	6.9	NR	NR		11.1	
Assadi [7]	135	3.7–7.4	NR	62		15.5	
Derlin [8]	385	6–7.4	6–8	48		NR	
Gafita [9]	1000	NR	NR	22		11.6	
Khreish [10]	215	6.9	NR	65		12	
Khreish [11]	539	6.5	6	57		12	
Maffey [12]	NR	6	6–10	50		12	
Paganelli [13]	56.5	3.7–5.5	10	31		NR	
Rasul [14]	72	7.4	4	58		28	
Rathke [15]	59	NR	8	35		NR	
Sathekge [16]	57	6	8	70		18	
Seifert [17]	363	7.5					
Seifert [20]	NR	6/7.5	7.5	44		12	
Violet [21]	190	7.5	8	64		13.3	
Yadav [22]	333	7.8–8.7	NR	45.5		14	
Yadav [23]	221	NR	NR	39		17	
Yordanova [24]	208	6.2	7.5	NR		17	
Zacherl [25]	112	7.8 MBq	8	50		NR	
Aghdam [26]	95	5.7	NR	45.4		NR	
Barber [27]	120	6.3	NR	48		18	
Grubmuller [28]	61	7.4	4	47.4		24	
Gupta [29]	143	7.4	NR	22.7		NR	
Heck [30]	165	7.4	6–10	38		12	
Kessel [31]	294	6.2		25		9.9	
McBean [32]	137	5.9	NR	45		NR	
Sathekge [33]	NR	7.5 (MBq)		88		NR	
Soydal [34]	260	6	6–8	33		12	
Suman [35]	NR	4.4–5.6	10–12	42.5		12	
Van Kalmthout [36]	200	6	6	57		11.3	
Von Eyben [37]	23	4.6	8	80		>30	
Yordanova [38]	208	6.1	NR	40		12	
Zhang [39]	60	6.4	8	44		15	
Kesavan [40]	20.5	5.5	8	40		NR	
Rahbar [41]	361	6.1	8	33		14	
Ahmadzadehfar [42]	206	NS	8	38		15	
Brauer [43]	NS	6.1	NR	53		8	

Abbreviations: NR: not reported.

**Table 2:** Clinical characteristics and prediction of OS.

Clinical Characteristic		Publications	Number of Patients	Meta-Analytic *p* Values
Patients	Previous chemotherapy	[6,25,27,34]	321	2.8 × 10^−6^
	Performance status	[4,6]	536	1.4 × 10^−6^
Cancer	Site of metastases	[5,27]	343	7.1 × 10^−5^
	Serum alkaline phosphatase	[38,41,43]		4.1 × 10^−4^
PRLT	Second series of PRLT	[37,38]	75	4.5 × 10^−4^
Response	PSA decline ≥ 50%	[4,30,31,34,41,42]	480	1.5 × 10^−10^
